# The rapid detection of a neonatal unit outbreak of a wild-type *Klebsiella variicola* using decentralized Oxford Nanopore sequencing

**DOI:** 10.1186/s13756-025-01529-2

**Published:** 2025-02-07

**Authors:** Rhys T. White, Michelle Balm, Megan Burton, Samantha Hutton, Jamaal Jeram, Matthew Kelly, Donia Macartney-Coxson, Tanya Sinha, Henrietta Sushames, David J. Winter, Maxim G. Bloomfield

**Affiliations:** 1 Health Group, Institute of Environmental Science and Research, Porirua, 5022 New Zealand; 2 Department of Microbiology and Molecular Pathology, Awanui Labs Wellington, Wellington, 6021 New Zealand; 3 Infection Prevention and Control, Te Whatu Ora/Health New Zealand, Capital, Coast & Hutt Valley, Capital, Wellington, 6021 New Zealand

**Keywords:** Genomics, Disease outbreaks, Phylogenetic analyses, Infection control, Multilocus sequence typing

## Abstract

**Background:**

*Klebsiella variicola* has been implicated in neonatal intensive care unit (NICU) outbreaks previously and can be misidentified as *Klebsiella pneumoniae*. An increased incidence of *K. pneumoniae* bacteremia on the NICU of our institution was notified to the infection prevention and control (IPC) team in May 2024. The four isolates involved displayed wild-type susceptibility, so had not been detected via multidrug-resistant organism surveillance. This triggered investigation with a nanopore-based decentralized whole-genome sequencing (dWGS) system in operation at our laboratory.

**Methods:**

Since early 2022, the hospital laboratory at Wellington Regional Hospital has been performing dWGS using the Oxford Nanopore MinION device. This allows for prospective genomic surveillance of certain hospital-associated organisms, but also rapid reactive investigation of possible outbreaks. Isolates are sequenced in the hospital laboratory and undergo multilocus sequence typing (MLST). If transmission events are suspected, sequence data are transferred to the reference laboratory, the Institute for Environmental Science and Research (ESR) for high-resolution bioinformatic analysis.

**Results:**

Within 48 h of notification isolates had been subcultured and sequenced. This showed that three of four isolates were in fact *K. variicola*, and two of these were sequence type (ST)6385. This sequence type had not been seen previously at our institution, so transmission was suspected. Environmental sampling revealed *K. variicola* ST6385 in two sink traps on the unit, and prospective sequencing of all *K. pneumoniae* isolates from NICU samples revealed two further infants with *K. variicola* ST6385. Subsequent phylogenetic analysis at ESR using original sequence data showed tight clustering of these isolates, confirming an outbreak. Sink traps were disinfected, environmental cleaning procedures were updated, and a strict focus on hand hygiene was reinforced on the ward. No further isolates were detected, and the outbreak was closed after two months.

**Conclusions:**

Access to dWGS at the level of the local hospital laboratory permitted rapid identification of an outbreak of an organism displaying no unusual antimicrobial resistance features at a point where there were only two known cases. This in turn facilitated a rapid IPC response.

**Supplementary Information:**

The online version contains supplementary material available at 10.1186/s13756-025-01529-2.

## Background

*Klebsiella variicola* is an emerging pathogen with a broad host range, causing infections in humans, animals, plants, while persisting in hospitals and other environments [1–3]. The *K. variicola* genome can harbor numerous virulence factors and antimicrobial resistance (AMR) determinants [4]. Additionally, the *K. variicola* genome can contain genes involved with nitrogen fixation, plant colonization, and mutualistic insect relationships [4]. These features enable widespread environmental persistence of *K. variicola*, potentially serving as a reservoir for AMR across diverse ecological niches [4].

Identifying *K. variicola* can be problematic for clinical microbiology laboratories because some commercial systems misidentify it as *Klebsiella pneumoniae*, leading to under-recognition [5, 6]. The clinical relevance of this misidentification is uncertain. However, some evidence suggests *K. variicola* is associated with more severe bloodstream infection than *K. pneumoniae* [7], and there are concerns regarding its propensity for spreading AMR [4, 6]. Notably, the prevalence of IncF plasmids, particularly the IncFIB_K_ subtype, in *K. variicola* raises concerns due to their role in disseminating extended-spectrum beta-lactamases (ESBLs), carbapenemases, and colistin resistance genes, posing significant challenges for treatment and infection control efforts [8]. *K. variicola* has been implicated in nosocomial infection and hospital outbreaks, including several reports in neonatal intensive care units (NICUs), suggesting its potential for nosocomial transmission like other *Klebsiella* species [9–11].

In early 2022 the hospital laboratory at Wellington Regional Hospital (WRH) established a decentralized whole-genome sequencing (dWGS) system using Oxford Nanopore sequencing to support local hospital infection prevention and control (IPC) [12]. In this context ‘decentralized’ refers to sequencing and initial bioinformatics analysis performed in the hospital diagnostic laboratory, with sequence data transferred to a central reference laboratory for high-resolution bioinformatic analysis if initial results suggest possible transmission events [13]. The Genomics and Bioinformatics Team and the Antibiotic Reference Laboratories at the Institute of Environmental Science and Research (ESR) serves the function of the reference laboratory for WRH. A decentralized model was adopted because the hospital laboratory lacks the on-site bioinformatics expertise for fine-scale WGS analysis.

In May 2024, WRH NICU observed an increased incidence of invasive *K. pneumoniae* infections, prompting further investigation using dWGS. This revealed that several isolates were actually *K. variicola*, belonging to sequence type (ST)6385, a lineage not previously reported in clinical settings. Here we describe the rapid genomic investigation of this outbreak.

## Methodology

### Setting

WRH provides tertiary services to a population of approximately 500,000 in the lower North Island/Te Ika-a-Māui of New Zealand/Aotearoa. The WRH NICU is a Level 3 facility resourced for 37 infants, providing specialized medical and surgical care for extremely premature infants (as early as 23 weeks gestation) and those requiring ventilation, intravenous feeding, and other intensive care support. The ward has six multi-cot rooms, each with two sinks, and four single isolation rooms.

Awanui Labs Wellington (an International Accreditation New Zealand ISO15189 accredited medical laboratory) provides on-site clinical diagnostic services to WRH and the surrounding region. The microbiology and molecular departments handle around 300,000 samples yearly. Identification of cultured bacterial isolates is performed using the Vitek^®^ MS PRIME (bioMérieux) with database v3.3.0. Phenotypic susceptibility testing of Enterobacterales is performed using the Vitek^®^ II instrument (bioMérieux) and the AST-N311 card, according to the European Committee on Antimicrobial Susceptibility Testing (EUCAST) guidelines [14].

### Decentralized whole-genome sequencing program

In early 2022, Awanui Labs Wellington established a prospective dWGS program [12]. Sequencing is performed weekly, allowing for both ‘proactive’ and rapid ‘reactive’ sequencing. ‘Proactive’ refers to the sequencing of certain IPC target organisms (e.g., *Clostridioides difficile*) in real-time as they are identified in the laboratory [15]. ‘Reactive’ refers to the sequencing of organisms suspected by IPC to be involved in hospital transmission events, which was the case with the outbreak presented here.

Details on DNA extraction are available in the Supplementary Materials. For isolates sequenced before March 2023, library preparation used 50 ng of genomic DNA with the Oxford Nanopore Technologies rapid barcoding kit 96 (SQK-RBK110-96) as per the manufacturer’s instructions. The library was loaded onto a R9.4 flow cell (FLO-MIN106) and run on an Oxford Nanopore MinION device for 20–40 h with MinKNOW v22.10.10. After March 2023, including during the outbreak, libraries were created using 50–100 ng of genomic DNA (prepared with the rapid barcoding kit 96 (SQK-RBK114-96)), and sequenced on an R10.4.1 flow cell (FLO-MIN114) with MinKNOW v23.04.5. The initial bioinformatic analysis performed in the hospital laboratory is determination of a multilocus sequence type (MLST) using Krocus v1.0.3 [16] with default settings to query the raw FASTQ files against the *Klebsiella* PasteurMLST sequence definition database [17] hosted on BIGSdb v1.47.0 [18].

### Genome annotation for kv240612_barcode19

As part of this investigation, and in keeping with our decentralized approach, sequence data were transferred to the Genomics and Bioinformatics Team at ESR for further analysis. In the absence of an available ST6385 reference genome, we selected kv240612_barcode19, a clinical isolate from a neonatal blood culture, as our reference genome (index case on the WRH NICU identified in May 2024). The FASTQ files outputted from the basecalling were corrected with single-read error correction using the HERRO algorithm (using the ‘dorado correct’ function using model-v1, dorado v0.7.2) [19]. HERRO-corrected reads underwent *de novo* assembly using Flye v2.9.2 [20] using a genome size estimate of 5.5 Mb and three polishing iterations. The assembly representing strain kv240612_barcode19 was annotated using Prokka v1.14.6 [21]. Prophage regions were identified using PHASTER [22] and then annotated using Pharokka v1.6.1 [23]. Mobile genetic elements were identified using IslandViewer 4 [24] and ISsaga v2.0 [25] (ISfinder platform [26]), followed by manual curation using Artemis v18.2.0 [27].

### Retrospective genome analyses

During 2022–2023, before this outbreak, one of the target organisms for proactive dWGS was *K. pneumoniae*, whereby all isolates from hospital inpatients were systematically sequenced. On further analysis of the sequence data, many of these isolates were identified as *K. variicola* and were therefore used to genomically contextualize the current outbreak. Original Fast5 sequence files were converted to Pod5 using pod5 v0.3.2 (https://github.com/nanoporetech/pod5-file-format, accessed on 01 August 2024) and then basecalled using Dorado v0.7.2 (https://github.com/nanoporetech/dorado, accessed on 01 August 2024). The base-calling process was carried out using the ‘super accuracy’ models, with a batch size of 2008 and a chunk size of 1000, while other parameters were kept at their default settings. Further details of laboratory methods including: nanopore read quality control, genome assembly, MLST, and virulence and antibiotic resistance gene genotyping, are available in Supplementary Materials.

### Taxonomic classification of the *K. variicola* genomes

To identify the nearest taxonomic neighbors to the suspected *K. variicola* genomes, the assemblies were input into a Genome Taxonomy Database Toolkit (GTDB-Tk) genome-based taxonomy (GTDB-Tk v2.1.1 with GTDB package R207_v2 [28]). Only sequences within the genus *Klebsiella* and *Escherichia* (g_, genus) were extracted from the GTDB-Tk and used to construct a concatenated reference alignment of 117 bacterial marker genes. The taxonomic tree was inferred using maximum-likelihood approximation with FastTree v2.1.7 [29] under the WAG model [30] of protein evolution with gamma-distributed rate heterogeneity [31] (+ GAMMA).

### Assembly-based variant detection and *K. Variicola* species-level phylogeny

A core-genome alignment was generated from the 50 local *K. variicola* genomes using Parsnp v1.7.4 [32] with the kv240612_barcode19 chromosome (GenBank: CP165787) serving as the reference to call single-nucleotide variants (SNVs). Resulting SNV alignments were used to reconstruct phylogenies. A maximum-parsimony tree was reconstructed using the heuristic search feature of PAUP v4.0a [33] before adding 1,000 bootstrap replicates. A maximum-likelihood phylogenetic tree was reconstructed using RaxML v8.2.12 [34] (GTR-GAMMA correction) by optimizing 20 distinct, randomized maximum-parsimony trees before adding 1,000 bootstrap replicates. The resulting phylogenetic trees were visualized using FigTree v1.4.4 (http://tree.bio.ed.ac.uk/software/figtree/, accessed on 01 August 2024).

### High-resolution read-mapping approach to variant calling and SNV density analysis

The quality trimmed and filtered reads for the five other outbreak-associated *K. variicola* ST6395 isolates were aligned to the reference kv240612_barcode19 chromosome using minimap2 v2.24 [35, 36] configured with the ‘map-ont’ preset for long-read data. The resulting Sequence Alignment/Map (SAM) file was converted to Binary Alignment/Map (BAM) format using SAMtools v1.20 [37]. The BAM file was sorted and indexed to facilitate downstream analysis. The median and range of sequence read data depth were calculated using the ‘mpileup’ tool from SAMtools, with the ‘-aa’ flag and mapping quality of ≥ 1. Variant calling was conducted using Clair3 v1.0.8 [38] with the ‘r1041_e82_400bps_sup_v500’ model, and flags ‘--include_all_ctgs’, ‘--no_phasing_for_fa’, and ‘--haploid_precise’. Variants were filtered using BCFtools v1.20 [39] to exclude those with a quality score below 30. To visualize SNV density across the kv240612_barcode19 reference chromosome, we used the ggplot2 v3.4.0 library [40] package in the R package v4.2.2 [41]. A histogram was plotted with binwidth set to 100 base pairs to represent SNV density along the genome. Consensus sequences for each of the five other ST6385 genomes were created by integrating strain-specific SNVs into the kv240612_barcode19 reference chromosome. A multiple-pseudo-genome alignment (5,500,654 bp) was then input into the Gubbins algorithm v3.2.1 [42] (default settings, “raxml mode” with the General Time Reversible (GTR) GAMMA correction) to identify recombination.

## Results

### Outbreak response

**Fig. 1:**
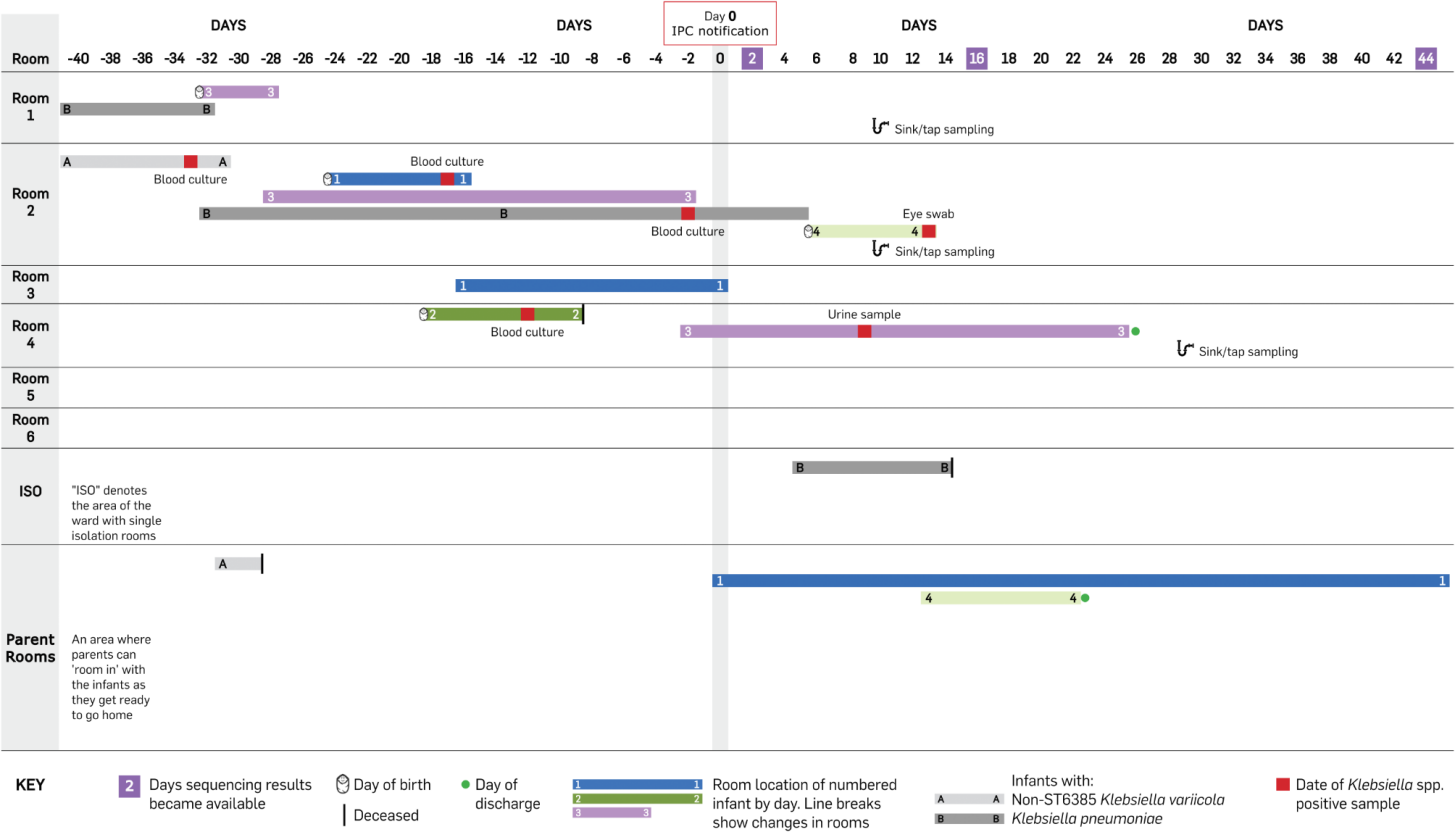
Line chart of the *Klebsiella variicola* ST6385 outbreak in the neonatal intensive care unit (NICU). The x-axis represents days, with day 0 being the day the hospital infection prevention and control (IPC) team was alerted by the NICU of four cases of *Klebsiella* spp. associated bacteremia over the preceding month (actual dates are omitted for patient privacy). Each horizontal shaded bar represents the days spent by the numbered infant in the room as indicated on the y-axis. Infants with non-ST6385 *K. variicola* (lighter-shaded grey bars), or *Klebsiella pneumoniae* (darker-shaded grey bars) are labelled with letters instead of numbers. Infant A was born on day − 76 and infant B was born on day − 63

In late May 2024, the IPC team were alerted by the NICU of four cases of *K. pneumoniae* bacteremia over the preceding month. These isolates displayed wild-type phenotypic antimicrobial susceptibility (resistant to amoxicillin only), so had not been identified via multidrug-resistant organism surveillance. This represented an increase above baseline incidence, so the isolates were retrieved and subcultured on the same day. The following day they were added to the weekly sequencing run, which was brought forward. By the next day, less than 48 h after notification, initial results showed that three of the four isolates were actually *K. variicola*, and two of these were ST6385. ST6385 had not been seen at the hospital previously, so transmission was suspected. Following this, any *K. pneumoniae* isolated from infants in NICU (or recent discharges) were prospectively sequenced. This revealed two further infants colonized with *K. variicola* ST6385 (Fig. 1). One of the infants with *K. variicola* bacteremia died shortly after the infection was detected. The remaining three infants with *K. variicola* ST6385 recovered and were eventually discharged. Following initial mitigations, no further *K. variicola* isolates were identified within the unit and the outbreak was closed after two months.

Environmental sampling was carried out on sinks/faucets in the three rooms infants with *K. variicola* had spent the most time in, and the two ultrasound machines used on the unit (Fig. 1, and Supplementary Materials for methods). Sinks/faucets were sampled due to proximity to bed spaces of affected infants, while ultrasound machines were sampled because the outbreak spanned several rooms and these machines were the primary shared equipment used between rooms. *K. variicola* ST6385 was isolated from two of six sink-trap water samples, with all other remaining environmental samples *K. variicola* negative. As part of the response, cleaning schedules for the ultrasound machines were reviewed and updated, additional cleaning of cot spaces was implemented, and affected sink traps were disinfected using a quaternary ammonium compound. Strict adherence to Standard Precautions was emphasized for clinical care for all infants. The outbreak totaled four infants, two associated with bacteremia (Table 1).

**Table 1:** Genomic characteristics of *Klebsiella variicola* strains from a neonatal outbreak in Wellington, New Zealand

Sample ID	Collection date	Source	Sample type	MLST	Capsule locus	Lipopolysaccharide locus	SRA accession no.
kv240612_barcode18	Apr 2024	Human	Blood	ST3938	KL71	O3/O3a	SRR30084997
kv240612_barcode19	May 2024	Human	Blood	ST6385	Untypeable	O3/O3a	SRR30084996
kv240612_barcode20	May 2024	Human	Blood	ST6385	Untypeable	O3/O3a	SRR30084995
kv240612_barcode24	Jun 2024	Human	Urine	ST6385	Untypeable	O3/O3a	SRR30084994
kv240612_barcode25	Jun 2024	Sink	-	ST6385	Untypeable	O3/O3a	SRR30084993
kv240709_barcode65	Jun 2024	Human	Eye swab	ST6385	Untypeable	O3/O3a	SRR30084992
kv240709_barcode69	Jun 2024	Sink	-	ST6385	Untypeable	O3/O3a	SRR30084991

MLST, multilocus sequence type; SRA, Sequence Read Archive; ST, Sequence Type

### A high-quality reference genome was generated for the index *K. Variicola* ST6385 case

Sequencing of kv240612_barcode19 initially produced 48,767 single-ended reads with a median read length of 4,223 bp (N50 = 26,672 bp) and a median read quality score of 21.0. HERRO correction refined this to 23,193 single-ended reads (444.5 total megabases) with a median length of 13,593 bp (N50 = 27,418 bp).

*De novo* assembly revealed a 5,500,654 bp circular chromosome with 57.42 % GC content (Supplementary Materials, Figure S1a). Using HERRO-corrected reads, the chromosome was sequenced to a median depth of 75× (range: 44 to 641×). Multiple antibiotic resistance genes were present, although no clinically relevant phenotypic resistance was observed (Supplementary Materials, Table S1). Virulence factors included *fim* and *mrk* operons (fimbrial adhesins), *Klebsiella* K and O loci (capsule and O-antigen synthesis), and iron acquisition systems (*iro*E, *iut*A, and iron-enterobactin operon). The capsule polysaccharide locus was not typable due to an insertion flanked by IS*Ecl1* and IS*Ec33* downstream of *wcaJ* (Supplementary Materials, Figure S2). The lipopolysaccharide locus was typed as O3/O3a serotype. The chromosome also contained several efflux pump systems (*aae*RXAB, *emr*BAR, *emr*AB-OMF, *mar*ORAB-Ea, *mdt*ABCD-*bae*SR, *acr*AB), and metal resistance operons (*ars*BCR, *cus*ABFCRS). Additional genomic features included five prophage regions, a genomic island integrated into *thr*R, and a type VI secretion system.

The complete genome of kv240612_barcode19 included the plasmid pkv240612_barcode19A (169,125 bp, FIB plasmid), carrying several key resistance and transport systems (Supplementary Materials, Table S1). Using HERRO-corrected reads, the plasmid was sequenced to a median depth of 145× (range: 129 to 311×). pkv240612_barcode19A harbored the *sil*E gene encoding a silver-binding protein, and the *cus*ABFCRS operon, both contributing to putative silver resistance. Putative copper resistance was conferred by the *cop*ABCD operon. The plasmid also carried the *ars*RBCH operon for arsenic resistance. Additionally, two transport systems were identified: the *urt*ABCDE operon, an ABC-type urea permease, and the *fec*IRABCDE operon for iron uptake.

### *K. Variicola* ST6385 is genomically distinct from other local *K. variicola*

We analyzed 58 *K. variicola* genomes from isolates collected in 2022 (*n* = 39), 2023 (*n* = 11), 2024 (*n* = 1), and throughout the outbreak (*n* = 7) (Supplementary Materials, Table S2). Of the 58 genomes, eight were excluded: five due to insufficient sequencing depth (< 15×) and three due to genome lengths outside the extended interquartile range (i.e., 5,261,825 bp and 6,092,438 bp) (Supplementary Materials, Table S3). After quality control, 50 genomes were retained for further analysis (Supplementary Materials, Table S4). The analyzed genomes were predominantly from 2022 (*n* = 34) and 2023 (*n* = 8), with one from 2024, and the seven outbreak isolates. The *de novo* assembly quality metrics for these genomes are presented in the Supplementary Materials (Table S5).

**Fig. 2:**
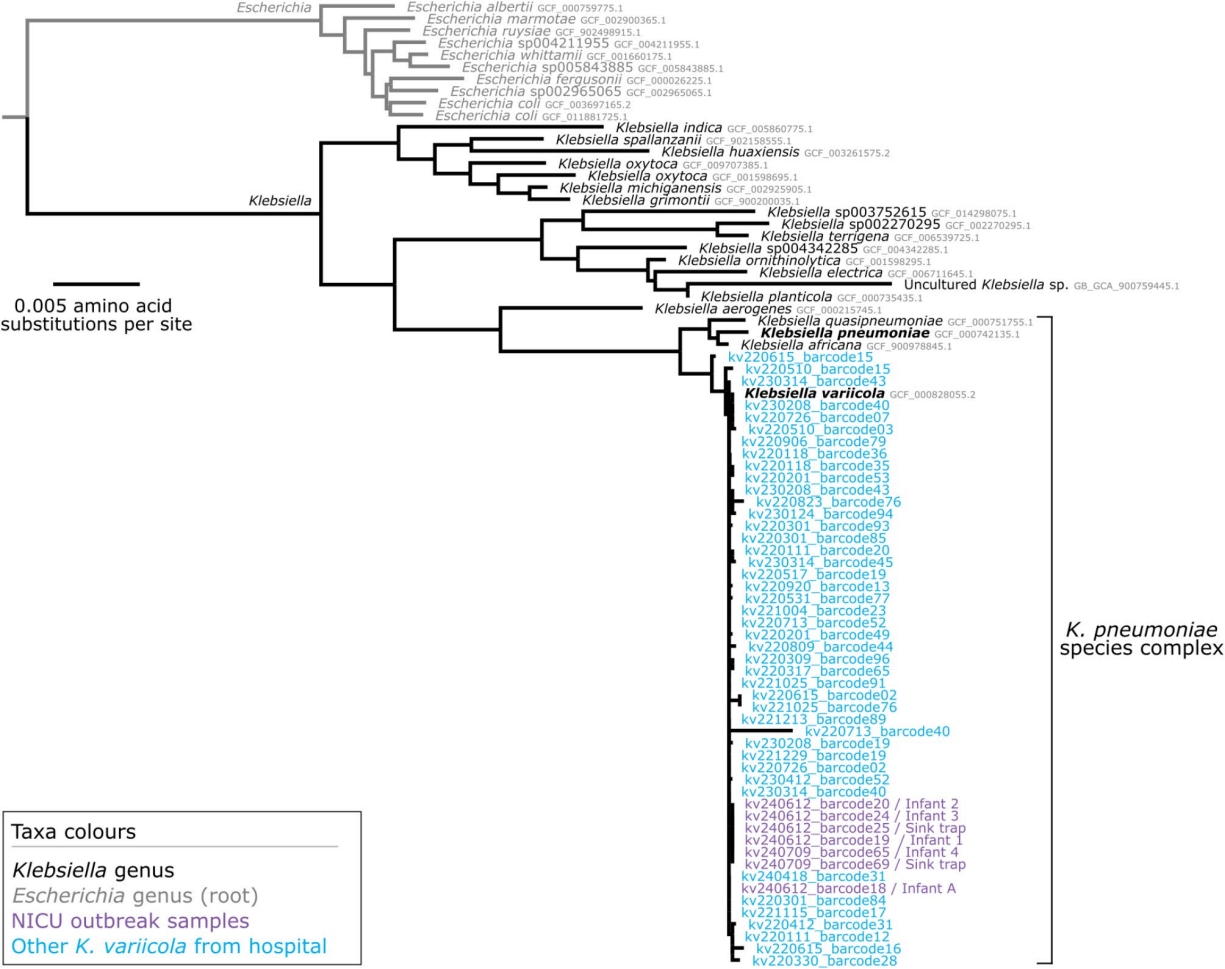
Taxonomic tree of the genus *Klebsiella*. The maximum-likelihood approximation was reconstructed using a concatenated alignment of 117 conserved bacterial markers. Taxonomy is shown according to the GTDB (s__, species) from NCBI taxonomy. GenBank assembly accession numbers are displayed after the genome names. The blue taxon represents the strain sequenced in this report. The tree is rooted according to the outgroup species *Escherichia coli*

**Fig. 3:**
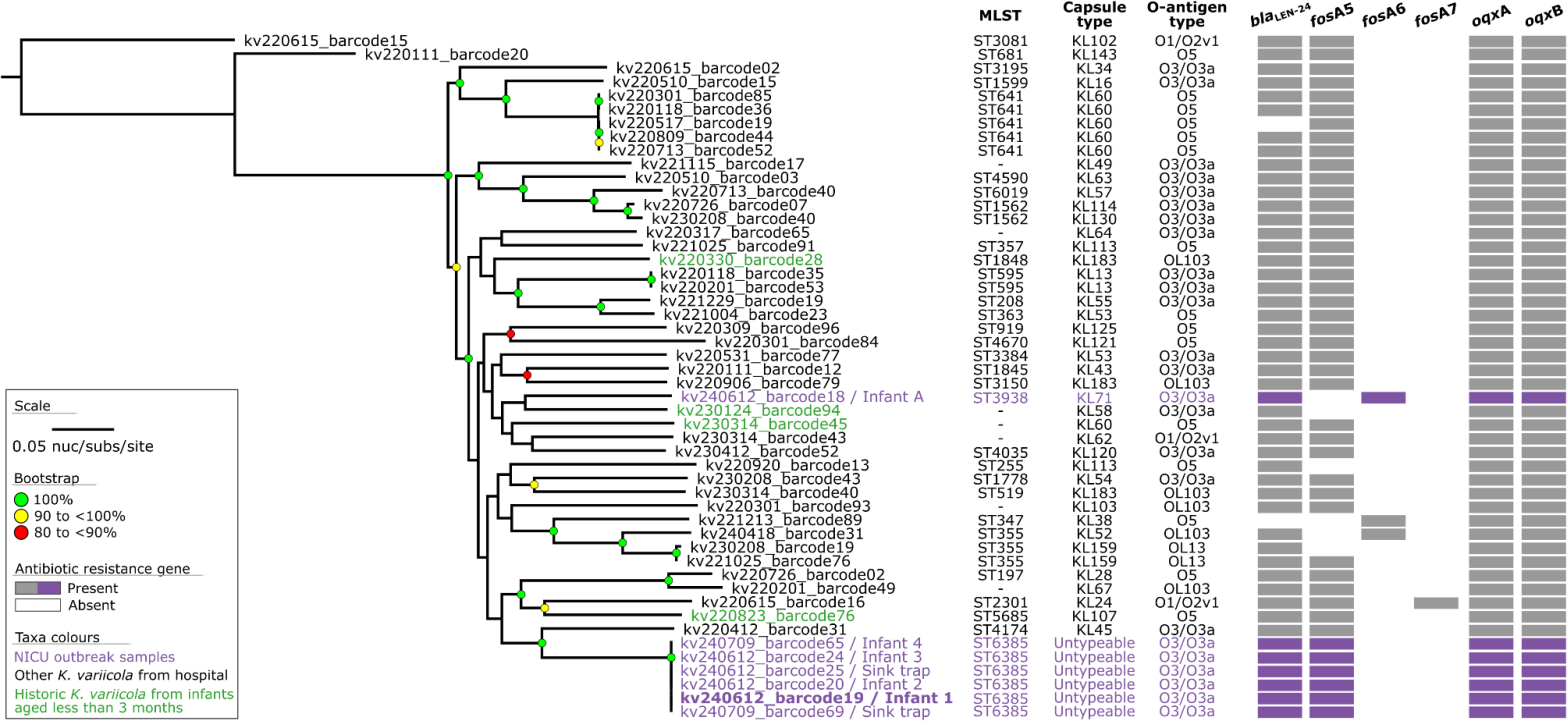
Maximum-likelihood phylogeny of *Klebsiella variicola* isolates from Wellington Regional Hospital. The phylogeny was inferred from 178,995 core-genome single-nucleotide variants (SNVs) from 50 genomes. SNVs were derived from a core-genome alignment of 4,283,084 bp and were called against the chromosome of sample kv240612_barcode19 (GenBank: CP165787). The phylogenetic tree is rooted according to the kv220615_barcode15 outgroup. Bootstrap values > 80 % (1,000 replicates) are shown

The GTDB-tk taxonomy analysis confirmed that these 50 genomes clustered with *K. variicola* and not *K. pneumoniae* (Fig. 2). Following this, the core-genome analysis identified 178,995 SNVs across the 50 genomes (Fig. 3). These SNVs were called against the chromosome of isolate kv240612_barcode19 using assembly-based variant detection (see Methods). A maximum-likelihood phylogenetic tree was constructed and rooted at genome kv220615_barcode15 (Fig. 3; see branch lengths expressed as SNVs in Supplementary Materials, Figure S3), corresponding to the root of *Klebsiella africana* (RefSeq: GCF_900978845), *K. pneumoniae* (RefSeq: GCF_000742135), and *Klebsiella quasipneumoniae* (RefSeq: GCF_000751755) (Fig. 2). The genome for isolate kv240612_barcode18 was classified as ST3938, which is genomically distinct from the other *K. variicola* genomes collected in this outbreak. The remaining six genomes, representing isolates from four infants and two sink traps, were classified as ST6385 and formed a distinct cluster. The long branch leading to the ST6385 cluster shows these six genomes are genomically distinct from previously sequenced *K. variicola* strains from the NICU, indicating a common origin. The six outbreak isolates were also sent to an affiliated laboratory that uses the Bruker MALDI Biotyper^®^ (Bruker Daltonik, v9 of the MBT 8468 database) which correctly identified all isolates as *K. variicola.*

### Minimal SNV differences and recombination events across human- and sink-derived *K. variicola* ST6385 genomes

For the six outbreak isolates, the SNV differences compared to the index isolate (kv240612_barcode19, infant 1) were 0 (kv240709_barcode69, sink trap), 1 (kv240612_barcode25, sink trap), 5 (kv240709_barcode65, infant 4), 15 (kv240612_barcode24, infant 3) and 71 (kv240612_barcode20, infant 2). The larger number of SNV differences for the latter two isolates were located predominantly in the urea transport operon, with Gubbins analysis suggesting these were due to recombination events (Supplementary Materials, Figure S4), with minimal SNV differences otherwise.

## Discussion

In previous investigations, we have shown the value of proactive dWGS for early identification of hospital outbreaks [15, 43]. Here we demonstrate the value of reactive dWGS for rapidly identifying and precisely defining an outbreak. Within 48 h of the initial notification, rapid MLST analysis performed at the local laboratory level provided a sufficient degree of suspicion to trigger IPC investigations and actions, whilst simultaneously excluding two unrelated cases from the outbreak. This revealed further affected infants, and an environmental reservoir of the organism, which could then be addressed. Furthermore, the outbreak was detected when there were only two known cases and the organism displayed no distinctive AMR or other phenotypic features, highlighting the high level of precision rapid dWGS can bring to IPC investigations. Had rapid dWGS not been available it seems likely that this outbreak would have progressed unnoticed to a larger outbreak with further invasive infections. Having access to dWGS in the hospital laboratory gives a high degree of flexibility to respond promptly to local IPC needs. In this case, communication between IPC and the laboratory meant isolates could be retrieved immediately, the weekly sequencing run could be brought forward, and actionable results produced in a short timeframe, with high-resolution off-site genomic analyses following later. Had isolates themselves needed to be shipped to the reference laboratory for sequencing and analysis this speed and agility of response would not have been possible. Many hospital laboratories have sufficient molecular experience to perform sequencing but do not have access to expert bioinformaticians for high-resolution WGS analysis, a major advantage of a dWGS model.

Organisms in the genus *Klebsiella*, predominantly *K. pneumoniae*, are important and well-described NICU pathogens [44], with the ability to transmit between infants, cause severe nosocomial infection and harbor key AMR genes [45]. More recently, several reports have implicated *K. variicola* as a cause of clinical infection and outbreaks among neonates [9–11]. This investigation supports the importance of *K. variicola* in the NICU setting and serves as a reminder that it may be misidentified as *K. pneumoniae*. The organism in this outbreak displayed a propensity for invasive infection and environmental persistence. Several identified virulence factors, such as outer membrane protein A, K locus for capsule synthesis (albeit novel in our study), outer lipopolysaccharide O antigen, and siderophores, are similar to those previously described in hypervirulent *K. pneumoniae* [46, 47]. However, while these factors are associated with virulence, the strain in this outbreak does not meet the full criteria for hypervirulence, such as the presence of specific genes (e.g., *iuc*A, *rmp*A) [46]. As such, further phenotypic investigations are required to confirm hypervirulence.

The organism also harbored genes associated with environmental persistence (through biofilm formation) [48], including heavy metal tolerance, efflux pumps, type 1 and 3 fimbriae, and a type VI secretion system. Furthermore, plasmid-borne elements may have enhanced the isolate’s metal resistance profile and environmental nutrient acquisition, such as iron uptake systems. These features likely support bacterial survival in hospital environments and may indirectly enhance virulence by facilitating infection establishment. The presence of a conjugative F plasmid *tra* operon (Figure S1b) indicates potential horizontal gene transfer, raising concerns about the dissemination of AMR and virulence-associated genes in the hospital setting. The ability of *K. variicola* to survive in the hospital environment is of IPC concern not only because of its innate virulence, but previous reports demonstrate an ability to acquire and disseminate plasmids containing important AMR genes in the NICU environment [10].

Recent reports have implicated sinks in the transmission of important hospital pathogens and questioned the role of sinks in healthcare [49]. There have been moves to create ‘waterless’ augmented care units including in neonatal settings [50]. Although it is unclear from our analysis the precise role sinks played in transmission on the unit, their ability to act as reservoirs for the outbreak organism was demonstrated. As part of our response, the affected sinks and traps were chemically disinfected, however recent evidence suggests that this may have limited effectiveness, albeit with a different compound [51]. This has generated ongoing discussions between the IPC and NICU teams regarding the need for sinks on the unit.

A limitation of this analysis was that we did not actively screen infants for the outbreak organism, and as such more infants than those identified may have been affected. This was due both to the lack of validated screening methods for this fully susceptible organism and the preference to utilize a unit-wide focus on strict hand hygiene and environmental cleaning as the core response. The environmental sampling was also performed opportunistically, rather than using specific environmental sampling techniques such as touch plates. As such, the sensitivity of sampling may have been compromised and other environmental sources of the organism missed. Had further invasive infections occurred, systematic screening of infants and the environment may have been necessary.

## Conclusions

In conclusion, this analysis has demonstrated the ability of decentralized whole-genome sequencing to provide rapid outbreak detection to facilitate timely infection prevention and control action. Speed and agility are key factors of an outbreak response, and in this case likely prevented further cases in a highly vulnerable population. This investigation revealed that *Klebsiella variicola*, often misidentified as *Klebsiella pneumoniae*, can cause severe disease and transmission in the NICU setting, with environmental persistence particularly in sink traps.

## Electronic supplementary material

Below is the link to the electronic supplementary material.

Supplementary Material 1

## Data Availability

DNA sequence data are available in the National Center for Biotechnology Information (NCBI) BioProject accession numbers: PRJNA1142676 (2022 samples), PRJNA1142678 (2023 samples), and PRJNA1142680 (2024 samples). The raw Oxford Nanopore Technologies (ONT) sequence reads are available under accession numbers: SRR30085170 to SRR30085208 (2022 samples), SRR30087320 to SRR30087330 (2023 samples), SRR30084991 to SRR30084998 (2024 samples). The complete assembly for strain kv240612_barcode19 has been deposited to GenBank under the accession numbers CP165787 and CP165788.

## Abbreviations

AMR: Antimicrobial resistance
dWGS: Decentralized whole genome sequencing
ESBLs: Extended-spectrum beta-lactamases
ESR: Institute for Environmental Science and Research
EUCAST: European Committee on Antimicrobial Susceptibility Testing
GTDB-Tk: Genome Taxonomy Database Toolkit
HERRO: Haplotype-aware ERRor cOrrection
IPC: Infection prevention and control
MLST: Multi locus sequence typing
NICU: Neonatal intensive care unit
SNV: Single-nucleotide variant
ST: Sequence type
WRH: Wellington Regional Hospital
