# Supplementary material for: The rapid detection of a neonatal unit outbreak of a wild-type Klebsiella variicola using decentralized Oxford Nanopore sequencing
Source: Antimicrob Resist Infect Control. 2025 Feb 7;14:6. doi: 10.1186/s13756-025-01529-2 (PMC11806699; doi:10.1186/s13756-025-01529-2)
Supplement: Supplementary file 1 — Supplementary Material 1 [file 13756_2025_1529_MOESM1_ESM.pdf]

SUPPLEMENTARY APPENDIX

The rapid detection of a neonatal unit outbreak of a wild-type *Klebsiella variicola* using decentralized Oxford Nanopore sequencing

Authors

Rhys T. White[1], Michelle Balm[2,3], Megan Burton[2], Samantha Hutton[2], Jamaal Jeram[2], Matthew Kelly[3], Donia Macartney-Coxson[1], Tanya Sinha[2], Henrietta Sushames[3], David J. Winter[1], Maxim G. Bloomfield[2,3]

Affiliations

[1]Institute of Environmental Science and Research, Health Group, Porirua 5022, New Zealand

[2]Awanui Labs Wellington, Department of Microbiology and Molecular Pathology, Wellington 6021, New Zealand

[3]Te Whatu Ora/Health New Zealand, Infection Prevention and Control, Capital, Coast & Hutt Valley, Wellington 6021, New Zealand

Address for Correspondence

Corresponding author: Rhys White, Institute of Environmental Science and Research, Porirua, New Zealand; Telephone: +64-4-914-0700; E-mail: rhys.white@esr.cri.nz

<u>This file includes the following:</u>

Supplementary Methods.

Supplementary Figure S1. Major genomic elements in *Klebsiella variicola* strain kv240612_barcode12.

Supplementary Figure S2. Major structural features and nucleotide pairwise comparisons of the capsule loci in *Klebsiella variicola* sequence type (ST)6385 and ST3384.

Supplementary Figure S3. Maximum-parsimony phylogeny of *Klebsiella variicola* isolates from Wellington Regional Hospital.

Supplementary Figure S4. Single-nucleotide variant (SNV) distribution and genomic element comparison across human- and sink-derived *Klebsiella variicola* sequence type (ST)6385 genomes.

Supplementary Methods

Extraction of DNA

Organisms from clinical samples selected for sequencing were subcultured for purity onto 5% sheep blood agar and incubated for 24-48 hours at 35°C. DNA extraction for *Klebsiella* spp. isolates involves suspending a 10µL loop of bacteria in 250 µL of phosphate-buffered saline (PBS), incubating at 37°C for at least 30 min, and then extracting on the MagNA Pure 96 instrument A (Roche, Switzerland; DNA and Viral NA Small-Volume Kit with Pathogen-Universal-200 Extraction protocol), which was already in use in at Awanui Laboratories Wellington. Following extraction, the DNA samples were stored in the refrigerator at 4°C until the appropriate sequencing run. The length of storage time varied depending on when the samples were received relative to the next scheduled sequencing run.

Quality control for the nanopore sequence data

NanoStat v1.6.0 from the Nanopack v1.6.0 suite (*1*) was used for an initial quality assessment of the basecalled and adapter-trimmed nanopore reads (FASTQ). NanoFilt v2.8.0, also from NanoPack, was used for read trimming. Initially, 52 nucleotides were trimmed from the start and end of each read to remove low-quality regions from the reads. Subsequently, NanoFilt was used again to filter out reads with a quality score below Q7. For taxonomic profiling and the detection *K. variicola* in the filtered nanopore sequence data, we used Kraken v2.1.3 (*2*) with default parameters and an NCBI Reference Sequence (RefSeq) database (*3*), Standard (https://benlangmead.github.io/aws-indexes/k2, accessed on 01 August 2024). This database contained references for archaea, bacteria, human, viruses, plasmids, and the 'UniVec core' subset of the UniVec database (a database of vector, adaptor, linker, and primer sequences).

***de novo* assembly of the nanopore sequence read data**

Filtered nanopore sequence reads were *de novo* assembled using Flye v2.9.2 (*4, 5*) using a genome size estimate of 5.5 Mb and three polishing iterations. Subsequently, the filtered reads were aligned to the assembly using minimap2 v2.24 (*6, 7*), specifically configured for long-read data with the 'map-ont' preset. After the initial alignment, the assembly underwent a round of polishing to correct single nucleotide variants (SNVs) and insertions and deletions (INDELs) using racon v1.4.3 (*8*). Racon was set up with parameters: '--match 8' for match score, '--mismatch -6' for mismatch score, and '--gap -8' for gap penalty. To further refine the assembly, the filtered nanopore reads were realigned to the output from the first polishing round using

minimap2, followed by additional rounds of racon polishing. This process was repeated for two additional cycles (three rounds of racon polishing total). Each additional round involved aligning the filtered nanopore reads to the polished assembly, creating new alignment files (PAF format), and applying racon to correct the assembly. The parameters used for polishing were kept consistent throughout all cycles to ensure uniform error correction. After polishing with racon, the assemblies were further refined using medaka v1.8.0 (https://github.com/nanoporetech/medaka, accessed on 01 August 2024), using the 'super accuracy' models. The medaka corrected assembly was circularised using Circlator v1.5.1 (*9*). A final round of polishing was completed using medaka. The assembly metrics were assessed using QUAST v5.0.2 (*10*).

in silico genotyping of the outbreak-associated _K. variicola_ genome sequence data

in silico multi-locus sequence typing (MLST) was done using MLST v2.9 (https://github.com/tseemann/mlst, accessed on 01 August 2024) with default settings to query the assemblies against the *Klebsiella* PasteurMLST sequence definition database (*11*) hosted on BIGSdb v1.47.0 (*12*). The capsule polysaccharide (K) and lipopolysaccharide (O) types were determined using the command-line version of Kaptive v3.0.0b5 and the *Klebsiella* K or O locus databases (*13, 14*). ABRicate v1.0.1 (https://github.com/tseemann/abricate, accessed on 01 August 2024) was used to screen the assemblies for acquired antibiotic resistance genes using the ARG-ANNOT (*15*) database (last updated 15 September 2023).

Environmental sampling

For faucet sampling, distal aerators were removed, and a rayon-tipped bacterial swab (Copan Transystem™, Copan Diagnostics, California, United States) was passed around the proximal internal part of the aerator several times. The swabs were then placed in Amies transport medium and immediately transported to the laboratory. Upon arrival, the swabs were plated onto MacConkey Agar and inoculated into Brain Heart Infusion (BHI) broth. The MacConkey Agar plates were incubated at 35°C for five days, while the BHI broth was incubated at 35°C for 48 hours and then subcultured onto MacConkey Agar for an additional 72 hours. Colonies with morphological characteristics suggestive of *Klebsiella* species were isolated and identified using the Vitek® MS PRIME system (bioMérieux). If *Klebsiella* spp. were detected, the colonies were forwarded for sequencing, and non-*Klebsiella* colonies were disregarded.

For sink trap sampling, the traps were removed from the sinks, and the water present in each trap was collected into a sterile universal container and sent to the laboratory. Two 250 μL aliquots of the water were plated onto separate MacConkey Agar plates and incubated at 35°C for 48 hours. Any colonies exhibiting phenotypic characteristics consistent with *Klebsiella* spp. were isolated and identified using the Vitek® MS PRIME system (bioMérieux). Colonies identified as *Klebsiella* spp. were forwarded for sequencing, while non-*Klebsiella* colonies were excluded from further analysis.

For sampling ultrasound machine surfaces, two rayon-tipped bacterial swabs (Copan Transystem™, Copan Diagnostics, CA, USA) were used on each machine, pressed against various surfaces, including high-touch areas like the keyboard and probes, as well as less frequently touched parts. The swabs were then transported to the laboratory, where they were plated onto MacConkey Agar and incubated at 35°C for 48 hours. Suspicious colonies suggestive of *Klebsiella* spp. were isolated and identified using the Vitek® MS PRIME system (bioMérieux). Any *Klebsiella* spp. identified were sent for sequencing, and non-*Klebsiella* colonies were excluded from further analysis.

Supplementary Figures

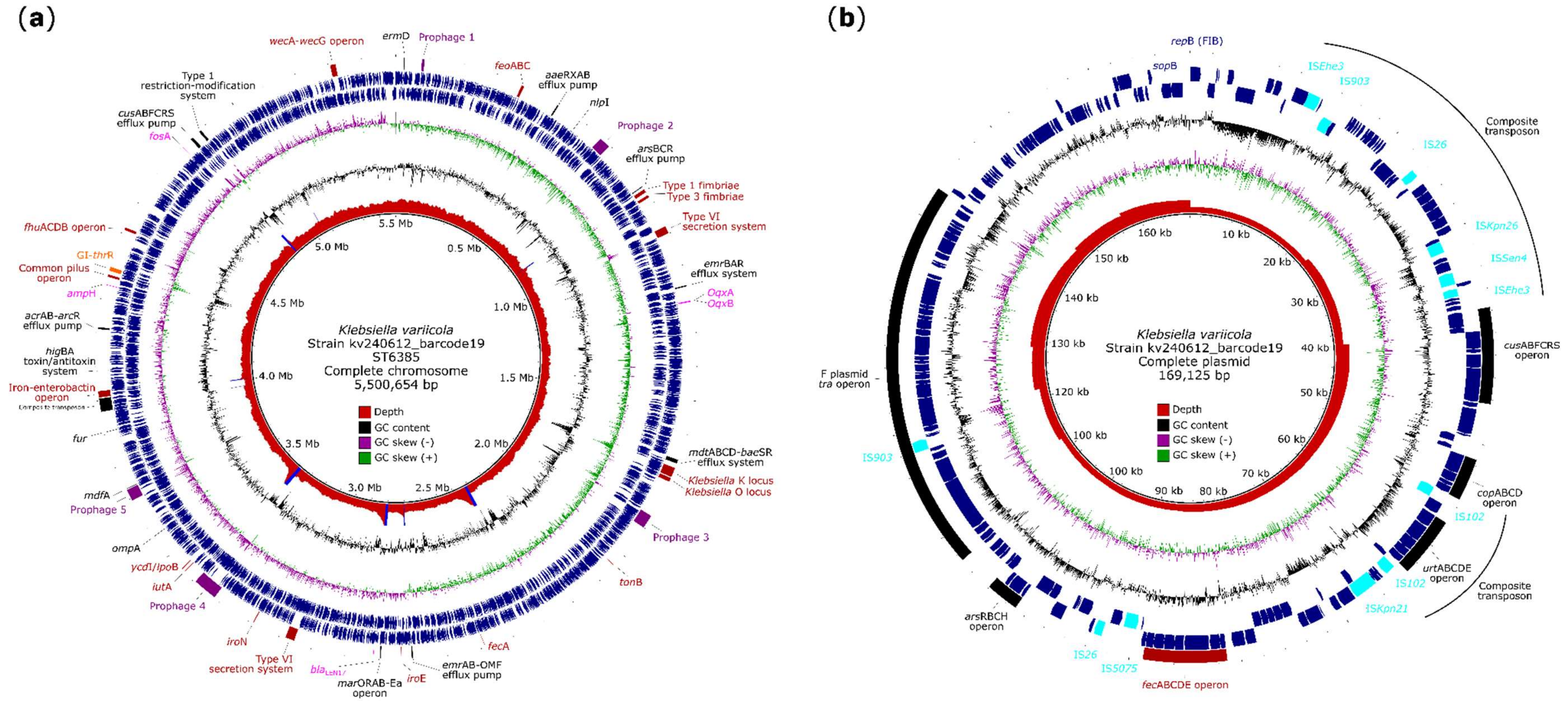

Supplementary Figure S1. Major genomic elements in *Klebsiella variicola* strain kv240612_barcode12. Circular representation of the *K. variicola* strain kv240612_barcode12 chromosome (a) and plasmid (b). The four innermost circles represent genome coordinates relative to kv240612_barcode12, genome coverage of mapped HERRO-corrected nanopore reads (graph maximum value 200× in a (blue indicated depth greater than 200×) and 350× in b), GC content, and GC skew. Rings 5 and 6 represent the coding sequences encoded on the reverse and forward strand, respectively. The outermost ring describes the positions of regions of interest (black), loci associated with virulence (red), genomic islands (orange), and prophages (purple). Antibiotic resistance genes are coloured pink. Image created using BRIG v0.95 (16).

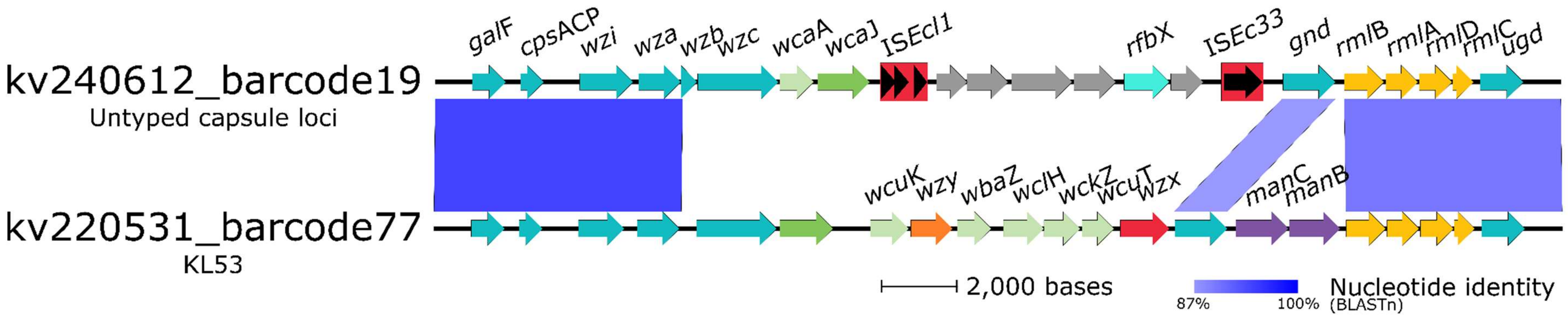

Supplementary Figure S2. Major structural features and nucleotide pairwise comparisons of the capsule loci in *Klebsiella variicola* sequence type (ST)6385 and ST3384. Nucleotide comparisons between the ST6385 genome (kv240612_barcode19) and the ST3384 genome (kv220531_barcode77), highlight differences between an unprecedented capsular region, and the known KL53. Blue shading between the K loci indicates nucleotide identity between sequences according to BLASTn (87 to 100%). Key genomic regions are illustrated as follows: Insertion Sequence (IS) elements (red box: IS*Ecl1* from IS*3* family and IS*Ec33* from *IS630* family), common proteins including core assembly machinery (blue arrows), *wcaJ* initiating glycosyltransferase (green arrow), other sugar synthesis and processing (light green arrows), putative O-antigen transporter *rfb*X (cyan), *wzx* flippase (red arrow), *wzy* capsule repeat unit polymerase (orange arrow), GDP-D-mannose synthesis (purple arrows), dTDP-L-rhamnose synthesis (yellow arrows), and hypothetical proteins (grey arrows). Image created using Easyfig v2.2.5 (*17*).

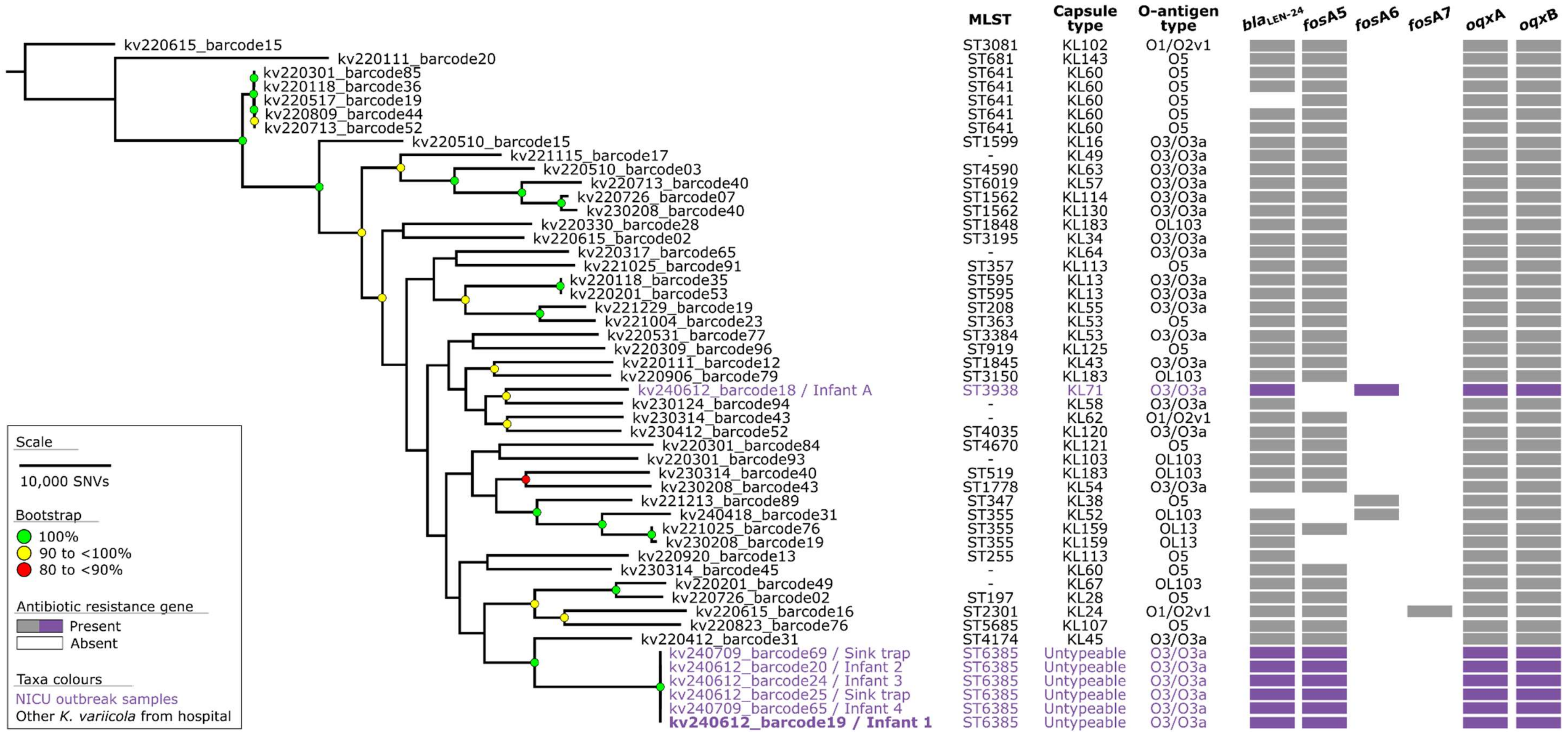

Supplementary Figure S3. Maximum-parsimony phylogeny of *Klebsiella variicola* isolates from Wellington Regional Hospital. The phylogeny was inferred from 178,995 core-genome single-nucleotide variants (SNVs) from 50 genomes. SNVs were derived from a core-genome alignment of 4,283,084 bp and were called against the chromosome of sample kv240612_barcode19 (GenBank: CP165787). The consistency index for the tree was 0.31. The phylogenetic tree is rooted according to the kv220615_barcode15 outgroup. Bootstrap values >80% (1,000 replicates) are shown.

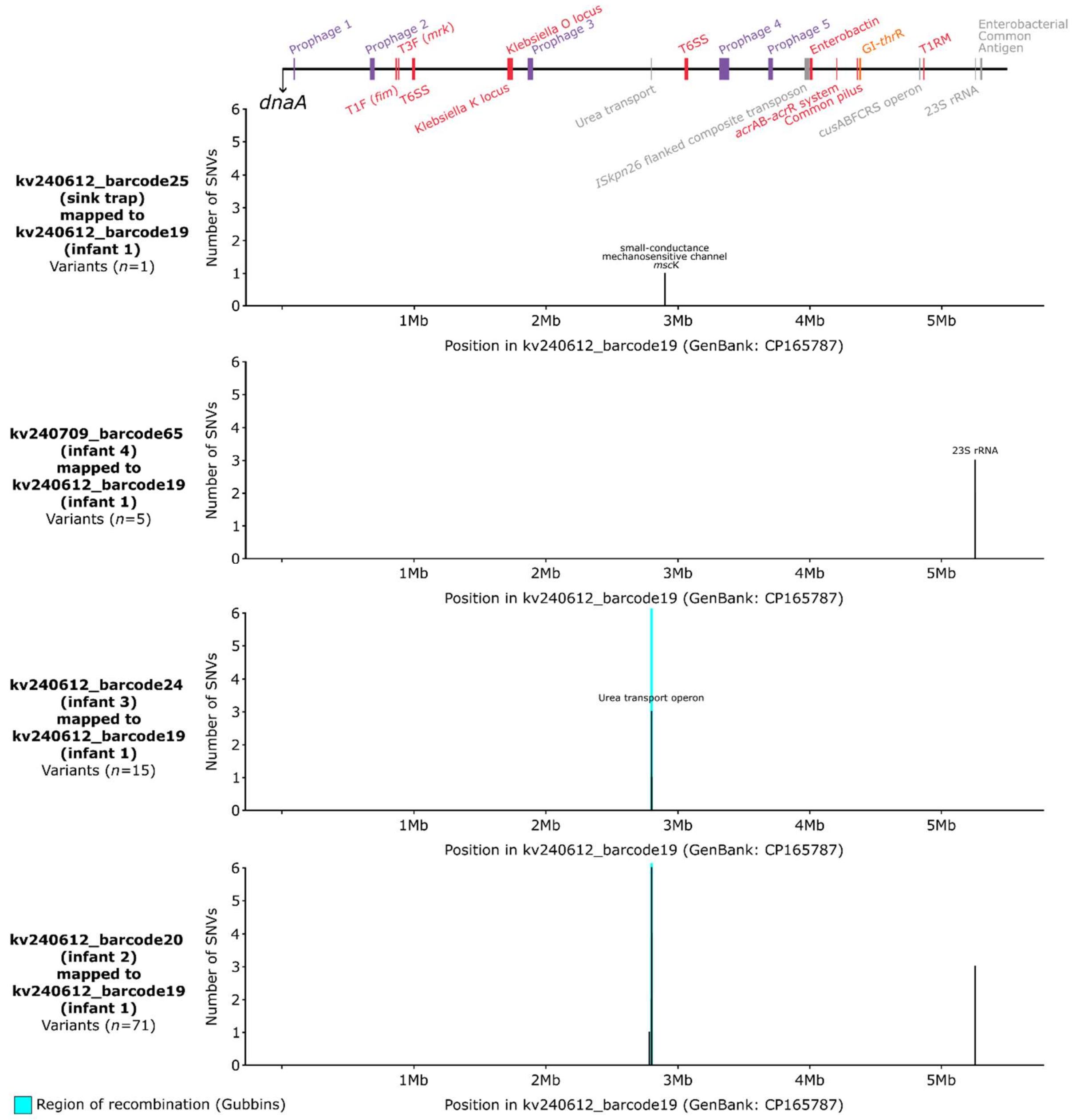

Supplementary Figure S4. **Single-nucleotide variant (SNV) distribution and genomic element comparison across human- and sink-derived *Klebsiella variicola* sequence type (ST)6385 genomes**. The distribution of SNVs and identification of genomic elements were compared across several genomes, including human-derived kv240612_barcode19, sink-derived kv240612_barcode25, and other human-derived genomes (kv240709_barcode65, kv240612_barcode24, and kv240612_barcode20). All genomes start from the gene encoding the chromosomal replication initiator protein DnaA. SNVs are displayed as total counts within a 100 bp sliding window. T1F, type 1 fimbriae; T3F, type 3 fimbriae; T6SS, type VI secretion system; T1RM, Type I restriction-modification; rRNA, ribosomal ribonucleic acid.

References

1. De Coster W, D'Hert S, Schultz DT, Cruts M, Van Broeckhoven C. NanoPack: visualizing and processing long-read sequencing data. Bioinformatics. 2018;34:2666-2669 DOI: 10.1093/bioinformatics/bty149

2. Wood DE, Salzberg SL. Kraken: ultrafast metagenomic sequence classification using exact alignments. Genome Biology. 2014;15:R46 DOI: 10.1186/gb-2014-15-3-r46

3. Sayers EW, Barrett T, Benson DA, Bryant SH, Canese K, Chetvernin V, et al. Database resources of the National Center for Biotechnology Information. Nucleic Acids Research. 2009;37:3124 DOI: 10.1093/nar/gkn741

4. Lin Y, Yuan J, Kolmogorov M, Shen MW, Chaisson M, Pevzner PA. Assembly of long error-prone reads using de Bruijn graphs. Proceedings of the National Academy of Sciences of the United States of America. 2016;113:E8396-E8405 DOI: 10.1073/pnas.1604560113

5. Kolmogorov M, Yuan J, Lin Y, Pevzner PA. Assembly of long, error-prone reads using repeat graphs. Nature Biotechnology. 2019;37:540-546 DOI: 10.1038/s41587-019-0072-8

6. Li H. Minimap2: pairwise alignment for nucleotide sequences. Bioinformatics. 2018;34:3094-3100 DOI: 10.1093/bioinformatics/bty191

7. Li H. New strategies to improve minimap2 alignment accuracy. Bioinformatics. 2021;37:4572-4574 DOI: 10.1093/bioinformatics/btab705

8. Vaser R, Sović I, Nagarajan N, Šikić M. Fast and accurate *de novo* genome assembly from long uncorrected reads. Genome Research. 2017;27:737-746 DOI: 10.1101/gr.214270.116

9. Hunt M, Silva ND, Otto TD, Parkhill J, Keane JA, Harris SR. Circlator: automated circularization of genome assemblies using long sequencing reads. Genome Biology. 2015;16:294 DOI: 10.1186/s13059-015-0849-0

10. Gurevich A, Saveliev V, Vyahhi N, Tesler G. QUAST: quality assessment tool for genome assemblies. Bioinformatics. 2013;29:1072-1075 DOI: 10.1093/bioinformatics/btt086

11. Diancourt L, Passet V, Verhoef J, Grimont PA, Brisse S. Multilocus sequence typing of *Klebsiella pneumoniae* nosocomial isolates. Journal of Clinical Microbiology. 2005;43:4178-4182 DOI: 10.1128/jcm.43.8.4178-4182.2005

12. Jolley KA, Bray JE, Maiden MC. Open-access bacterial population genomics: BIGSdb software, the PubMLST.org website and their applications. Wellcome Open Research. 2018;3:124 DOI: 10.12688/wellcomeopenres.14826.1

13. Wyres KL, Wick RR, Gorrie C, Jenney A, Follador R, Thomson NR, et al. Identification of *Klebsiella* capsule synthesis loci from whole genome data. Microbial Genomics. 2016;2:e000102 DOI: 10.1099/mgen.0.000102

14. Lam MMC, Wick RR, Judd LM, Holt KE, Wyres KL. Kaptive 2.0: updated capsule and lipopolysaccharide locus typing for the *Klebsiella pneumoniae* species complex. Microbial Genomics. 2022;8 DOI: 10.1099/mgen.0.000800

15. Gupta SK, Padmanabhan BR, Diene SM, Lopez-Rojas R, Kempf M, Landraud L, et al. ARG-ANNOT, a new bioinformatic tool to discover antibiotic resistance genes in bacterial genomes. Antimicrobial Agents and Chemotherapy. 2014;58:212-220 DOI: 10.1128/AAC.01310-13

16. Alikhan NF, Petty NK, Ben Zakour NL, Beatson SA. BLAST Ring Image Generator (BRIG): simple prokaryote genome comparisons. BMC Genomics. 2011;12:402 DOI: 10.1186/1471-2164-12-402

17. Sullivan MJ, Petty NK, Beatson SA. Easyfig: a genome comparison visualizer. Bioinformatics. 2011;27:1009-1010 DOI: 10.1093/bioinformatics/btr039
